# Safety and efficacy of aged garlic extract in dogs: upregulation of the nuclear factor erythroid 2-related factor 2 (Nrf2) signaling pathway and Nrf2-regulated phase II antioxidant enzymes

**DOI:** 10.1186/s12917-018-1699-2

**Published:** 2018-11-29

**Authors:** Osamu Yamato, Tadamitsu Tsuneyoshi, Mitsuyasu Ushijima, Hiroshi Jikihara, Akira Yabuki

**Affiliations:** 1 Laboratory of Clinical Pathology, Department of Veterinary Medicine, Joint Faculty of Veterinary Medicine, Kagoshima University, 1-21-24 Kohrimoto, Kagoshima, 890-0065 Japan; 2 Central Research Institute, Wakunaga Pharmaceutical Co. Ltd., 1624 Shimokotachi, Koda-cho, Akitakata-shi, Hiroshima, 739-1195 Japan; 3 Research Administration Department, Wakunaga Pharmaceutical Co. Ltd., 1624 Shimokotachi, Koda-cho, Akitakata-shi, Hiroshima, 739-1195 Japan

**Keywords:** Aged garlic extract (AGE), Dog, Nuclear factor erythroid 2-related factor 2 (Nrf2), Phase II antioxidant enzyme, NAD(P)H quinone dehydrogenase 1 (*NQO1*), Glutamate-cysteine ligase modifier subunit (*GCLM*)

## Abstract

**Background:**

Plants of *Allium* spp., including garlic (*A. sativum*) and onions *(A. cepa*), are known to be oxidatively toxic to canine erythrocytes resulting in Heinz body hemolytic anemia in dogs. In humans, these plants have been used as medicinal agents for multiple diseases since ancient times. Especially, fresh garlic extracted over a prolonged period produces less irritative and odorless aged garlic extract (AGE), containing unique and beneficial organosulfur compounds that can help prevent many kinds of diseases. In this study, the safety and efficacy of long-term oral administration of AGE is evaluated in dogs. The objectives are to confirm the safe dosage for long-term use and beneficial functions of AGE for dogs and to plan and design a canine health supplement or a preventive agent for multiple diseases based on the data of this study.

**Results:**

Beagles were orally administered AGE (45 or 90 mg/kg body weight once a day) or an equivalent amount of water as control for 12 weeks. In AGE-treated groups, at 12 weeks post-administration at a dose of 90 mg/kg, there were no observable changes in the clinical signs, complete blood count, and serum biochemical parameters. Heinz bodies and eccentrocytes, the markers of oxidative damage in erythrocytes, did not appear in blood smear examination. In order to further evaluate the beneficial effects of AGE on health of dogs, the expressions of nuclear factor erythroid 2-related factor 2 (Nrf2) gene (*NFE2L2*) and Nrf2-regulated phase II antioxidant enzyme genes (*NQO1*, *GCLM*, *HMOX1*, and *SOD2*) were determined in whole blood between pre- and post-AGE administration. The expression of *NFE2L2* gene was significantly upregulated in the AGE-treated groups [45 (*p* < 0.05) and 90 mg/kg (*p* < 0.01), 8 weeks] as compared to in the control group. Among the Nrf2-regulated enzymes examined, the expressions of *NQO1* [45 (*p* < 0.05) and 90 mg/kg (*p* < 0.01), 8 weeks] and *GCLM* [45 (*p* < 0.05) and 90 mg/kg (*p* < 0.01), 12 weeks] genes were significantly upregulated.

**Conclusion:**

The long-term oral administration of AGE at a dose of 90 mg/kg/day for 12 weeks did not show any adverse effects in dogs. Furthermore, the administration of AGE upregulated the gene expressions of canine Nrf2 and Nrf2-regulated phase II antioxidant enzymes. These results suggest that AGE might safely contribute to the health of dogs provided that the appropriate dosage is used.

**Electronic supplementary material:**

The online version of this article (10.1186/s12917-018-1699-2) contains supplementary material, which is available to authorized users.

## Background

Garlic (*Allium sativum*) has been used by humans since ancient times not only as food but also as a therapeutic agent for treatment of cardiovascular diseases, gastrointestinal disorders, bronchial asthma, and other disorders [1, 2]. Favorable biological and pharmacological properties of garlic that contribute to the treatment of such diseases and disorders have been documented in humans and rodents [3–7]. However, intake of either garlic or onion (*A. cepa*) causes hemolytic anemia, a potentially life-threatening event, in dogs due to the oxidation of erythrocytes that result in the formation of Heinz bodies and eccentrocytes [8, 9]. Therefore, the medicinal properties of these Allium plants have never been utilized for improvement in the health of dogs. It would be ideal if the medicinal properties could be utilized as a canine health supplement or a preventive agent for multiple diseases to maintain the health of dogs.

Fresh garlic extracted over a prolonged period produces a less irritative and odorless aged garlic extract (AGE) [6], which contains stable and water-soluble sulfur-containing amino acids, such as *S*-allylcysteine (SAC) and *S*-1-propenylcysteine (S1PC) [10–12]. Clinical studies on human subjects have demonstrated favorable pharmacological effects of AGE in atherosclerosis [13, 14], metabolic syndromes [15], and hypertension [16]. Numerous experimental studies have also revealed positive effects of AGE and its components, SAC and S1PC, in anti-oxidation [17], anti-aging [18, 19], immunomodulation [20, 21], anti-hypertensive [22, 23], anti-fatigue [24, 25], hepato-protective [26], anti-inflammatory [27, 28], and cardio-protective activities [29].

To examine the safety of AGE in animals, acute and 6-month-long administration studies were conducted in mice and rats, whose results suggested its safety nature; for example, AGE administration resulted in less gastric mucosal irritation as compared to when raw garlic was administered [30, 31]. However, to our knowledge, no studies have yet been conducted on the safety and efficacy of AGE in dogs. Therefore, this study was performed to evaluate the toxicity and safety of 12-week-long oral administration of AGE in dogs. Furthermore, amongst a variety of potential biological effects of AGE, nuclear factor erythroid 2-related factor 2 (Nrf2) signaling pathway and Nrf2-regulated phase II antioxidant enzymes were selected for evaluation of AGE efficacy on health of dogs, because the Nrf2 signaling pathway is expected to be a key to the multiple functions of AGE.

The objectives of this study are to confirm the safe dosage for long-term use and beneficial functions of AGE for dogs and to plan and design a canine health supplement or a preventive agent for multiple diseases based on the data of this study.

## Results

### Clinical outcome, food consumption, and body weight

All the dogs were kept active without any severe clinical deterioration during the study period. Most of the dogs were willing to drink AGE when it was fed. Coat condition and urine color were normal in all dogs during the experimental period.

Soft feces were observed in three dogs fed AGE (45 mg/kg), but not in any dogs that were fed AGE (90 mg/kg) and the control group (Additional file 1). The occurrence of soft feces was once to seven times in the three dogs over 84 days of the experimental period. Vomiting was observed four times in one dog fed AGE (45 mg/kg) and once in one dog fed AGE (90 mg/kg), but not in the control dogs (Additional file 1).

All the dogs consumed 230 g of dry food fed every day during the experimental period. There was no statistically significant difference in body weights among the three groups during the experimental period (Additional file 2)

### Hematology

There was almost no observable change in the data obtained from complete blood count (CBC) and blood smear examinations, except for a slightly significant change (*p* < 0.05) of the reticulocyte count between the group administered AGE at a dose of 45 mg/kg group and the control group a week after the administration (Table 1). Heinz bodies, eccentrocytes, and other types of poikilocytosis were not found even by an elaborate microscopic observation in any dog during the experimental period.

**Table 1:** Changes in the complete blood count and oxidative damage markers in erythrocytes in dogs fed aged garlic extract (45 mg/kg or 90 mg/kg body weight once a day) for 12 weeks

Parameters	Dose (mg/kg)	Week 0	Week 1	Week 4	Week 8	Week 12
RBC	0 (Control)	7.68 ± 0.49	7.59 ± 0.68	7.48 ± 0.59	7.18 ± 0.62	7.40 ± 0.86
(10^6^/μl)	45	7.14 ± 0.71	7.40 ± 1.14	7.32 ± 0.78	6.97 ± 0.72	7.36 ± 0.59
	90	7.38 ± 0.42	7.42 ± 0.64	6.93 ± 0.87	6.80 ± 0.80	7.34 ± 0.67
Hb	0 (Control)	17.0 ± 1.5	17.0 ± 2.3	16.5 ± 2.0	15.6 ± 1.9	16.4 ± 2.4
(g/dl)	45	16.3 ± 1.9	16.9 ± 2.8	16.4 ± 1.9	15.5 ± 1.7	16.6 ± 1.6
	90	16.3 ± 0.6	16.6 ± 0.7	15.4 ± 1.5	15.0 ± 1.2	16.3 ± 0.7
Ht	0 (Control)	50.6 ± 4.0	50.0 ± 5.9	49.0 ± 4.9	46.6 ± 5.1	47.8 ± 6.1
(%)	45	47.4 ± 5.6	48.9 ± 7.8	48.1 ± 5.3	45.8 ± 5.0	47.7 ± 5.5
	90	46.9 ± 2.2	47.2 ± 1.6	44.8 ± 3.3	43.7 ± 2.2	46.5 ± 1.8
MCV	0 (Control)	65.9 ± 4.3	65.9 ± 3.8	65.5 ± 3.8	64.9 ± 3.0	64.5 ± 2.7
(fl)	45	66.3 ± 1.9	66.1 ± 1.8	65.8 ± 2.5	65.7 ± 2.3	64.6 ± 2.6
	90	63.5 ± 2.7	63.9 ± 3.4	64.9 ± 3.5	64.6 ± 4.2	63.6 ± 3.9
MCH	0 (Control)	22.2 ± 1.1	22.3 ± 1.3	22.0 ± 1.2	21.8 ± 0.9	22.2 ± 0.8
(pg)	45	22.8 ± 0.8	22.9 ± 0.9	22.4 ± 0.8	22.3 ± 0.8	22.5 ± 0.5
	90	22.1 ± 0.8	22.5 ± 1.0	22.2 ± 0.8	22.1 ± 0.9	22.3 ± 1.1
MCHC	0 (Control)	33.7 ± 1.0	33.9 ± 1.1	33.6 ± 1.2	33.5 ± 1.1	34.4 ± 1.1
(g/dl)	45	34.4 ± 0.5	34.6 ± 0.5	34.1 ± 0.1	33.9 ± 0.1	34.8 ± 0.7
	90	34.7 ± 0.6	35.2 ± 0.5	34.4 ± 0.9	34.2 ± 1.1	35.2 ± 0.5
WBC	0 (Control)	10.9 ± 0.9	9.3 ± 0.5	9.8 ± 1.5	12.1 ± 1.7	11.2 ± 2.6
(10^3^/μl)	45	10.3 ± 0.8	9.8 ± 1.3	8.7 ± 1.2	10.4 ± 0.9	10.2 ± 0.7
	90	12.3 ± 1.2	9.9 ± 1.1	10.6 ± 1.5	11.8 ± 3.2	10.3 ± 1.9
PLT	0 (Control)	341 ± 62	331 ± 51	349 ± 55	355 ± 33	379 ± 47
(10^3^/μl)	45	346 ± 48	339 ± 41	348 ± 101	342 ± 82	355 ± 157
	90	325 ± 40	343 ± 125	357 ± 93	324 ± 45	336 ± 64
Reticulocyte	0 (Control)	8 ± 5	2 ± 1	7 ± 6	3 ± 1	3 ± 2
(‰)	45	9 ± 5	8 ± 2*	9 ± 6	7 ± 5	6 ± 3
	90	5 ± 1	4 ± 1	5 ± 3	3 ± 1	4 ± 1
Heinz body	0 (Control)	0	0	0	0	0
(‰)	45	0	0	0	0	0
	90	0	0	0	0	0
Eccentrocyte	0 (Control)	0	0	0	0	0
(‰)	45	0	0	0	0	0
	90	0	0	0	0	0

Data are presented as the mean ± standard deviation (n = 3). *RBC* erythrocyte count, *Hb* hemoglobin concentration, *Ht* hematocrit value, *MCV* mean corpuscular volume, *MCH* mean corpuscular hemoglobin, *MCHC* mean corpuscular hemoglobin concentration, *WBC* leukocyte count, *PLT* platelet count. *p < 0.05, compared with the water-treated control group

### Serum biochemistry

Among all the serum biochemical parameters examined, there was no tendency of increase or decrease on administration of AGE (Table 2). All the parameters were kept within the canine normal ranges. A slightly significant difference (*p* < 0.05) was observed in lactate dehydrogenase (LDH) activity between groups administered AGE (45 and 90 mg/kg) and the control group at weeks 4 and 8, in alkaline phosphatase (ALP) activity between the AGE (45 mg/kg)-administered and the control groups at week 12; and in total bilirubin (T-Bil) concentration between the AGE (90 mg/kg)-administered and the control groups before the administration. These significant changes did not result from the changes caused by AGE, but occurred due to the original difference for each individual within the normal ranges.

**Table 2:** Changes in serum biochemical parameters in dogs fed aged garlic extract (45 mg/kg or 90 mg/kg body weight once a day) for 12 weeks

Parameters	Dose (mg/kg)	Week 0	Week 1	Week 4	Week 8	Week 12
LDH	0 (Control)	49.2 ± 6.7	41.2 ± 0.5	73.8 ± 14.2	102.0 ± 39.0	72.0 ± 24.9
(U/l)	45	44.8 ± 5.7	44.2 ± 14.8	45.4 ± 11.4*	42.7 ± 8.5*	54.0 ± 14.4
	90	45.2 ± 9.3	32.2 ± 4.9	38.0 ± 2.4*	36.0 ± 8.7*	40.0 ± 10.4
ALT	0 (Control)	37.9 ± 5.7	39.2 ± 7.8	35.2 ± 4.9	35.0 ± 2.0	32.7 ± 4.7
(U/l)	45	47.8 ± 6.4	52.5 ± 20.6	43.9 ± 11.5	41.3 ± 10.2	34.7 ± 14.0
	90	35.6 ± 2.8	39.1 ± 8.9	32.3 ± 4.1	29.7 ± 5.1	35.3 ± 6.0
AST	0 (Control)	34.6 ± 9.3	31.1 ± 8.9	36.0 ± 13.1	38.0 ± 12.5	33.7 ± 13.2
(U/l)	45	33.4 ± 3.8	29.8 ± 4.4	33.7 ± 5.0	30.7 ± 5.5	30.3 ± 3.8
	90	33.2 ± 2.6	28.6 ± 1.2	29.6 ± 2.7	29.3 ± 0.6	28.3 ± 2.1
ALP	0 (Control)	108 ± 32	104 ± 34	97 ± 28	90 ± 21	84 ± 20
(U/l)	45	200 ± 56	198 ± 54	182 ± 45	164 ± 39	179 ± 43*
	90	141 ± 61	126 ± 53	150 ± 75	142 ± 78	121 ± 42
CK	0 (Control)	124 ± 17	110 ± 26	136 ± 25	162 ± 39	127 ± 28
(U/l)	45	113 ± 19	100 ± 14	112 ± 20	95 ± 26	111 ± 24
	90	125 ± 30	104 ± 36	104 ± 26	104 ± 20	100 ± 16
TP	0 (Control)	5.89 ± 0.43	6.13 ± 0.31	6.53 ± 0.19	6.04 ± 0.47	6.20 ± 0.58
(g/dl)	45	5.85 ± 0.14	5.94 ± 0.16	6.30 ± 0.40	5.86 ± 0.22	6.19 ± 0.18
	90	6.06 ± 0.60	6.31 ± 0.61	6.85 ± 0.50	6.29 ± 0.29	6.41 ± 0.40
Alb	0 (Control)	2.68 ± 0.27	2.67 ± 0.30	2.61 ± 0.24	2.45 ± 0.17	2.50 ± 0.18
(g/dl)	45	2.60 ± 0.22	2.52 ± 0.24	2.55 ± 0.20	2.41 ± 0.19	2.42 ± 0.28
	90	2.69 ± 0.17	2.61 ± 0.17	2.57 ± 0.18	2.34 ± 0.01	2.39 ± 0.06
Glb	0 (Control)	3.21 ± 0.27	3.47 ± 0.19	3.92 ± 0.07	3.59 ± 0.32	3.70 ± 0.42
(g/dl)	45	3.24 ± 0.09	3.42 ± 0.15	3.75 ± 0.55	3.45 ± 0.35	3.77 ± 0.46
	90	3.37 ± 0.44	3.69 ± 0.47	4.28 ± 0.35	3.95 ± 0.30	4.02 ± 0.45
Glu	0 (Control)	98.0 ± 2.3	100.2 ± 1.8	94.5 ± 2.4	91.0 ± 10.8	87.7 ± 6.8
(mg/dl)	45	110.2 ± 2.4	106.2 ± 7.2	103.7 ± 5.8	97.0 ± 6.2	96.3 ± 2.5
	90	103.9 ± 11.2	97.5 ± 7.1	93.1 ± 3.3	90.7 ± 2.5	91.3 ± 6.7
TG	0 (Control)	30.6 ± 8.5	35.8 ± 9.8	26.7 ± 1.6	29.3 ± 2.5	32.7 ± 7.0
(mg/dl)	45	29.0 ± 5.0	29.0 ± 5.0	30.2 ± 1.9	30.7 ± 6.5	30.7 ± 8.6
	90	33.0 ± 12.4	36.2 ± 8.3	30.2 ± 7.4	31.3 ± 5.7	30.0 ± 9.2
T-Cho	0 (Control)	136 ± 34	135 ± 26	124 ± 23	116 ± 24	141 ± 51
(mg/dl)	45	128 ± 14	126 ± 17	121 ± 19	117 ± 11	119 ± 9
	90	144 ± 43	147 ± 41	149 ± 41	135 ± 49	129 ± 45
BUN	0 (Control)	12.8 ± 2.4	14.0 ± 1.9	12.1 ± 0.7	11.9 ± 2.6	11.7 ± 3.4
(mg/dl)	45	13.2 ± 1.5	14.6 ± 1.9	13.2 ± 1.4	11.8 ± 0.6	13.0 ± 0.9
	90	12.1 ± 0.6	12.1 ± 0.4	12.5 ± 1.0	12.0 ± 0.6	11.4 ± 1.8
Cre	0 (Control)	0.62 ± 0.10	0.66 ± 0.10	0.66 ± 0.09	0.62 ± 0.11	0.61 ± 0.11
(mg/dl)	45	0.70 ± 0.09	0.70 ± 0.11	0.67 ± 0.05	0.62 ± 0.03	0.64 ± 0.04
	90	0.66 ± 0.12	0.64 ± 0.11	0.63 ± 0.08	0.59 ± 0.06	0.64 ± 0.06
T-Bil	0 (Control)	0.08 ± 0.01	0.08 ± 0.01	0.08 ± 0.01	0.07 ± 0.01	0.08 ± 0.01
(mg/dl)	45	0.10 ± 0.02	0.10 ± 0.03	0.10 ± 0.03	0.08 ± 0.02	0.10 ± 0.01
	90	0.12 ± 0.01*	0.09 ± 0.01	0.10 ± 0.02	0.07 ± 0.02	0.09 ± 0.02
CRP	0 (Control)	1.28 ± 0.90	1.63 ± 1.22	1.04 ± 0.87	0.78 ± 0.43	0.75 ± 0.43
(mg/dl)	45	1.44 ± 0.63	1.56 ± 0.70	0.85 ± 0.96	0.61 ± 0.56	0.74 ± 0.89
	90	0.92 ± 0.19	1.05 ± 0.41	1.02 ± 0.19	0.62 ± 0.15	0.44 ± 0.30
Ca	0 (Control)	9.65 ± 0.44	9.94 ± 0.24	9.83 ± 0.26	9.27 ± 0.15	9.50 ± 0.10
(mg/dl)	45	9.87 ± 0.31	9.89 ± 0.19	9.71 ± 0.51	9.30 ± 0.26	9.50 ± 0.36
	90	9.71 ± 0.17	9.70 ± 0.35	9.94 ± 0.43	9.23 ± 0.15	9.63 ± 0.15
iP	0	3.31 ± 0.31	3.47 ± 0.23	3.31 ± 0.27	3.03 ± 0.25	3.40 ± 0.20
(mg/dl)	45	3.40 ± 0.26	3.51 ± 0.44	3.61 ± 0.41	3.30 ± 0.44	3.63 ± 0.25
	90	3.40 ± 0.47	3.63 ± 0.39	3.56 ± 0.29	3.30 ± 0.26	3.33 ± 0.65
Na	0 (Control)	150.3 ± 3.0	152.3 ± 1.7	151.3 ± 1.9	145.7 ± 0.6	145.3 ± 0.6
(mEq/l)	45	154.4 ± 1.0	152.4 ± 1.6	151.3 ± 0.4	147.7 ± 0.6	145.3 ± 1.2
	90	151.0 ± 0.9	149.3 ± 0.6	150.8 ± 2.5	144.7 ± 1.5	145.0 ± 1.0
K	0 (Control)	4.81 ± 0.26	4.91 ± 0.24	5.07 ± 0.17	4.60 ± 0.14	5.05 ± 0.15
(mEq/l)	45	4.71 ± 0.33	4.92 ± 0.26	5.01 ± 0.09	4.55 ± 0.27	4.96 ± 0.17
	90	4.56 ± 0.35	4.82 ± 0.20	4.66 ± 0.34	4.44 ± 0.35	4.65 ± 0.35
Cl	0 (Control)	118.0 ± 2.4	118.3 ± 1.4	117.5 ± 0.6	113.7 ± 1.5	112.7 ± 1.5
(mEq/l)	45	118.9 ± 1.8	118.4 ± 2.0	118.2 ± 2.2	112.7 ± 1.5	112.0 ± 1.0
	90	119.3 ± 0.5	118.5 ± 0.6	119.4 ± 2.4	113.7 ± 1.5	113.7 ± 0.6

Data are presented as the mean ± standard deviation (*n* = 3). *LDH* lactate dehydrogenase, *ALT* alanine aminotransferase, *AST* aspartate aminotransferase, *ALP* alkaline phosphatase, *CK* creatine kinase, *TP* total protein, *Alb* albumin, *Glb* globulin, *Glu* glucose, *TG* triglyceride, *T-Chol* total cholesterol, *BUN* blood urea nitrogen, *Cre* creatinine, *T-Bil* total bilirubin, *CRP* C-reactive protein, *Ca* calcium, *iP* inorganic phosphorus, *Na* sodium, *K* potassium, *Cl* chloride. *p < 0.05, compared with the water-treated control group

### Gene expression of Nrf2 and Nrf2-regulated phase II antioxidant enzymes

**Fig. 1:**
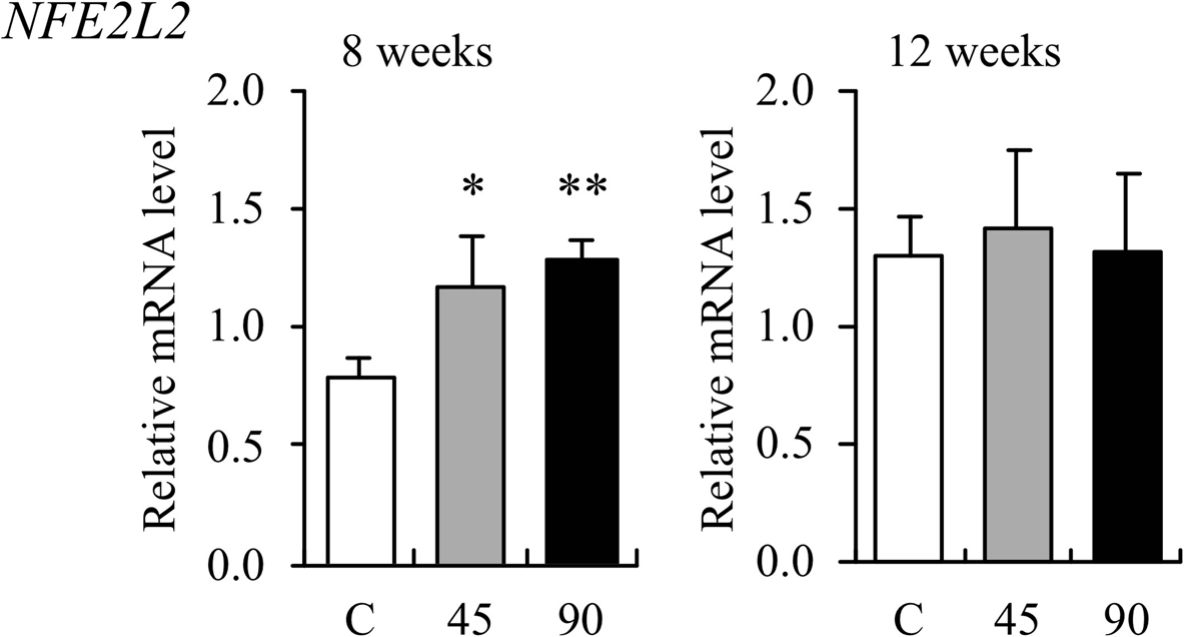
The effect of aged garlic extract (AGE) on the expression of canine *NFE2L2* gene in the whole blood. Relative mRNA expression levels in whole blood samples from dogs administered AGE at doses of 45 and 90 mg/kg/day at weeks 8 and 12 are shown as fold induction compared with those in water-treated control group (C). The expression level is calculated as the ratio of expression after administration to that before administration of AGE. Data are presented as the mean ± standard deviation of the results from three independent experiments. *p < 0.05 and **p < 0.01 vs control with a statistical significance

**Fig. 2:**
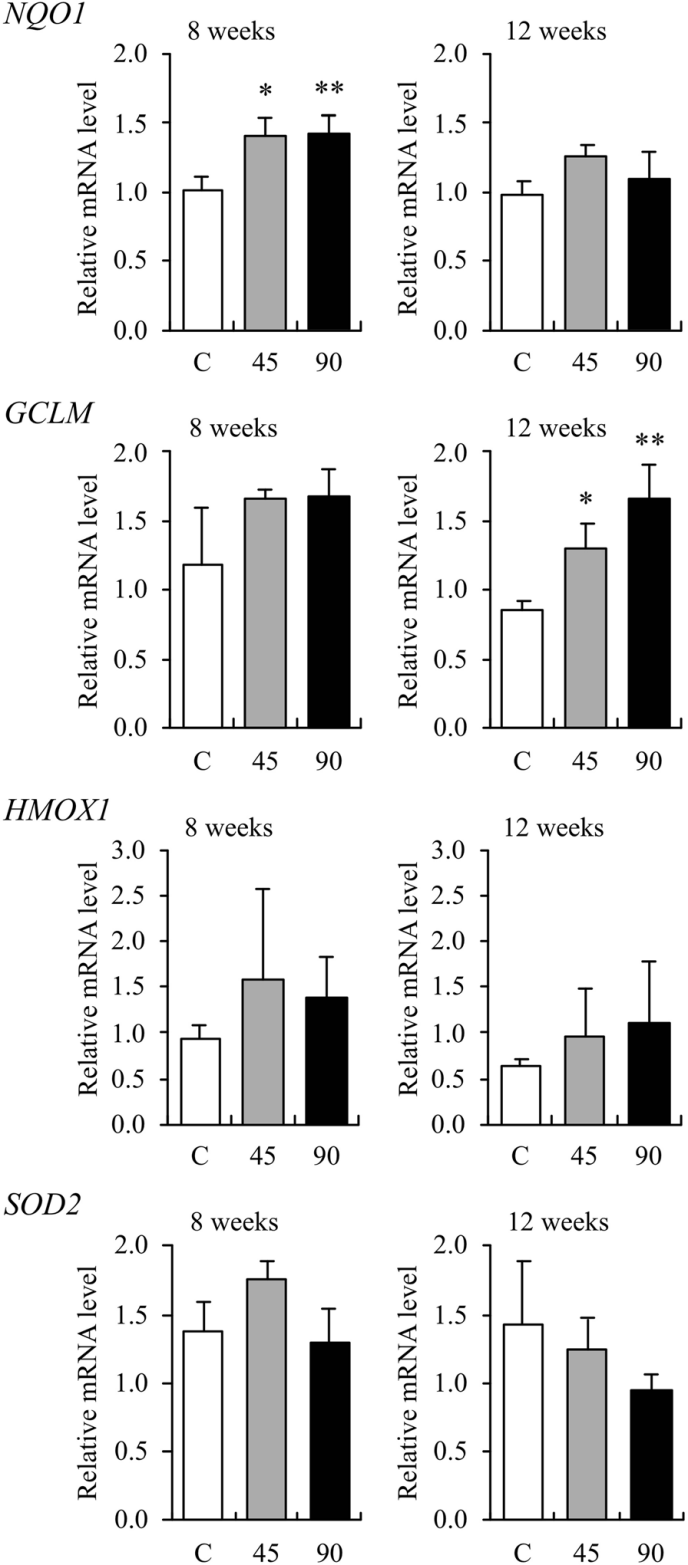
The effect of aged garlic extract (AGE) on the expression of canine *NQO1*, *GCLM*, *HMOX1*, and *SOD2* genes in the whole blood. Relative mRNA expression levels in whole blood samples from dogs administered AGE at doses of 45 and 90 mg/kg/day at weeks 8 and 12 are shown as fold induction compared with those in water-treated control group (C). The expression level is calculated as the ratio of expression after administration to that before administration of AGE. Data are presented as the mean ± standard deviation of the results from three independent experiments. *p < 0.05 and **p < 0.01 vs control with a statistical significance

The expression of Nrf2 gene (*NFE2L2*) increased significantly in the AGE [45 (*p* < 0.05) and 90 mg/kg (*p* < 0.01)]-administered groups compared to the control group after 8 weeks (Fig. 1). There was no statistical difference between the two AGE groups and the control group at week 12, although the gene expressions were slightly higher in the AGE groups than in the control group. Among the four Nrf2-regulated phase II antioxidant enzyme genes, the expressions of NAD(P)H quinone dehydrogenase 1 (*NQO1*), glutamate-cysteine ligase modifier subunit (*GCLM*), and heme oxygenase 1 (*HMOX1*) genes were higher in both the AGE groups than in the control group (Fig. 2). The expression of *NQO1* gene increased significantly in the AGE [45 (*p* < 0.05) and 90 mg/kg (*p* < 0.01)]-administered groups as compared to the control group at week 8. The expression of *GCLM* gene increased significantly only in the AGE [45 (*p* < 0.05) and 90 mg/kg (*p* < 0.01)]-administered groups as compared to the control group at week 12. The expression of superoxide dismutase 2 (*SOD2*) gene did not change on administration of AGE during the experimental period.

## Discussion

It has experimentally been demonstrated that the constituents of garlic have the potential to oxidize canine erythrocyte membranes and hemoglobin, inducing hemolysis associated with the appearance of eccentrocytes and Heinz bodies [9]. In addition, there have been a few cases of naturally occurring garlic-induced hemolytic anemia in canines [8, 32, 33]. Therefore, this has led to the opinion that foods containing garlic should not be fed to dogs [9]. Sodium 2-propenyl thiosulfate (2PTS) was identified to be present in boiled garlic as one of the causative agents of garlic-induced hemolytic anemia in dogs [34]. It can oxidize canine erythrocytes [34] and has the potential to induce Heinz body hemolytic anemia like one of the causative agents of onion-induced hemolytic anemia: sodium *n*-propyl thiosulfate (NPTS) [35–37].

In the present study, AGE was orally administered to dogs at a maximum dose of 90 mg/kg body weight once daily for long-term (12 weeks). The data on hematology (Table 1) and serum biochemistry (Table 2) showed no abnormal or toxic changes in CBC, hemolysis (reticulocytes, LDH, and T-Bil), erythrocyte oxidation (Heinz bodies and eccentrocytes), proteins [total protein (TP), albumin (Alb), and globulin (Glb)], hepatocyte damage [alanine aminotransferase (ALT) and aspartate aminotransferase (AST)], cholestasis [ALP, T-Bil, total cholesterol (T-Cho)], renal function [blood urea nitrogen (BUN) and creatinine (Cre)], muscular damage [creatine kinase (CK) and AST], glucose metabolism [glucose (Glu)], lipid metabolism [triglyceride (TG) and T-Cho)], acute phase protein [C-reactive protein (CRP)], minerals [calcium (Ca) and inorganic phosphorus (iP)], and electrolytes [sodium (Na), potassium (K), and chloride (Cl)], except for a few significant differences between each AGE-treated and the control groups due to each dog’s original value for a given variable within the normal ranges. The survival time of canine erythrocytes is estimated to be 100 days [38]. The administration period of AGE in the present study was approximately 84% of this survival time, when no Heinz bodies, eccentrocytes, or any other poikilocytes were detected in the blood smears from all the dogs. This suggests that continuous oral administration of AGE at a dose of 90 mg/kg does not cause anemia in normal dogs.

It is known that compounds formed from various precursors in fresh garlic, causes strong irritation to the gastric mucosa [10, 39], which can induce vomiting and diarrhea. It has been reported that AGE, compared with other processed garlic, causes less gastric irritation in dogs and rats [23, 39]. In the present study, soft feces or vomiting was temporarily observed in all dogs in the AGE (45 mg/kg)-administered group, but was observed just once in one dog at the 90 mg/kg dose. Therefore, these temporal soft feces and vomiting were presumably accidental occurrences in healthy dogs, and not associated with the administration of AGE. However, since the occurrence of these symptoms was slightly more prevalent in one dog (no. 6, Additional file 1) in the AGE (45 mg/kg)-administered group compared with that in other dogs, AGE may be associated with slight irritation of the gastrointestinal system in certain dogs. One of the most sensitive inflammation markers, CRP [40], did not increase in any of the dogs, suggesting that AGE does not stimulate inflammation of the canine gastrointestinal mucosa. Based on these clinical observations, AGE-based supplements or drugs for dogs should be designed together with certain mucoprotective agents. Furthermore, neither appetite reduction nor weight loss were observed in any of the dogs used in the present study.

These results from the evaluation of toxicity and safety of AGE demonstrate that the long-term oral administration of AGE at a dose of 90 mg/kg/day is completely safe in normal Beagles unlike intact garlic and other garlic preparations. Based on the safety of AGE in dogs, it is expected to not contain sodium alk(en)yl thiosulfates, including 2PTS and NPTS, which are oxidatively toxic to canine erythrocytes and have the potential to induce hemolytic anemia. However, Japanese and Korean dogs like Shiba Inus, Akita Inus, and Jindo dogs, include a special phenotype that usually shows no clinical manifestation, but these dogs are more susceptible to onion-induced hemolytic anemia and NPTS than normal dogs when the same doses of onions and NPTS are administered [35, 36, 41, 42]. Therefore, a lower-dose of AGE than 90 mg/kg/day should be considered safe in such dogs. Furthermore, cats are generally recognized as the species most susceptible to erythrocyte oxidation; this is partially because feline hemoglobin (Hb) molecule appears to be more susceptible to oxidative denaturation than does the Hb of other species [43], and thus, AGE should not be fed to cats without demonstration of the safe dose like in the present study.

Transcription factor, Nrf2, is a major regulator of cellular responses against environmental stresses [44, 45]. This factor induces the expressions of phase II antioxidant and detoxification enzymes after the exposure of environmental stresses. Kelch-like ECH-associated protein 1 (Keap1) is an adaptor subunit of Cullin 3-based E3 ubiquitin ligase, which acts as a sensor for oxidative and electrophilic stresses and subsequently regulates the activity of Nrf2. Without any environmental stress, Nrf2 is effectively ubiquitinated by the ligase activity of Keap1 and degraded rapidly in the proteasome system, resulting in the continuous suppression of the cellular Nrf2 activity. When cells are exposed to oxidative or electrophilic stresses, Keap1 loses its ability to ubiquitinate Nrf2, enabling Nrf2 to translocate to and accumulate in the nucleus. Accumulated intranuclear Nrf2 dimerizes with Maf proteins to form an Nrf2–Maf heterodimer that can bind the antioxidant response element or electrophile response element located in the regulatory regions of many cytoprotective enzymes on DNA, and finally activates its target genes. Well-known active compounds extracted from raw garlic, i.e., allicin, ajoene, and diallyl trisulfide, are demonstrated to produce their functions via Keap1–Nrf2 signaling pathway because these compounds have sulfur molecules which can deactivate Keap1 by reacting with its sulfhydryl groups [46–48]. However, these active compounds extracted from raw garlic should not be used for canine health because they have the potential to be oxidatively toxic to canine erythrocytes.

The AGE is highly bioavailable and exerts biological activity in both humans and experimental animals [10]. The process of aging gently modifies harsh and irritating compounds from raw garlic and naturally generates unique and beneficial compounds, such as SAC and S1PC through both enzymatic and natural chemical reactions. Both AGE and SAC perform their functions via Keap1–Nrf2 signaling pathway [10, 49], and S1PC is suspected to be an Nrf2 inducer. In the present study, the gene expressions of canine Nrf2 and Nrf2-regulated phase II antioxidant enzymes were analyzed to evaluate the potential multiple functions of AGE contributing to the health of dogs. As a result, among the four Nrf2-regulated phase II antioxidant enzyme genes, the expressions of *NQO1* and *GCLM* were observed to increase significantly by the long-term oral administration of AGE (Fig. 2). This suggests that AGE acts as an Nrf2 inducer and upregulates multiple genes related to antioxidation as well as detoxication via the Keap1–Nrf2 signaling pathway in dogs. The Nrf2 inducers in AGE might be SAC and S1PC, or other active compounds that have not been yet identified. Furthermore, the expression of *NFE2L2* gene increased significantly by the administration of AGE (Fig. 1). This may be due to a compensatory reaction to the decreased amounts of Nrf2 in the cytoplasm following its transport to the nucleus. Otherwise, AGE may contain certain compound(s) that can directly upregulate canine *NFE2L2* gene and result in an increase of Nrf2. In this case, AGE might upregulate the Keap1–Nrf2 signaling pathway in a synergetic manner.

## Conclusions

The long-term oral administration of AGE at a dose of 90 mg/kg/day for 12 weeks did not show any adverse effects in dogs. Furthermore, AGE administration upregulated the gene expressions of canine Nrf2 and Nrf2-regulated phase II antioxidant enzymes. These results suggest that AGE might safely contribute to the health of dogs provided that the appropriate dosage is used.

## Methods

### Preparation of AGE

The AGE was prepared by Wakunaga Pharmaceutical Co., Ltd. and was manufactured under license issued by the Ministry of Health and Welfare of Japan using the following steps: sliced raw garlic cloves were dipped into aqueous ethanol and extracted over 10 months at room temperature. The extract was dried at 65 °C for 25 h using a circulation dryer (HOH-A3, Takabayashi Rika Co., Ltd., Tokyo, Japan) with crystalline cellulose (CEOLUS UF-F702, Asahi Kasei Chemicals Corporation, Tokyo, Japan) to obtain a final AGE concentration of 33% (*w/w*). This product was pulverized into powder and stored at 4 °C until further use.

### Experimental animals and treatments

This experiment was conducted at the Research Institute for Animal Science in Biochemistry and Toxicology (Sagamihara, Kanagawa, Japan). Nine Beagles, aged 3 years with mean body weight of 11.6 kg (10.1–12.9 kg), were obtained from the Institute for Animal Reproduction (Kasumigaura, Ibaraki, Japan) and Kitayama Labes Co., Ltd. (Ina, Nagano, Japan) and returned to the original companies after this study. Before the experimentation, all the dogs were confirmed to be healthy based on physical examination, CBC, and serum biochemical analysis. The experimental facility was maintained at a temperature of 23 °C under a 12-h light–dark cycle (light 7 am–7 pm). Before and during the study, each dog was fed 230 g of dry food (TC-1, Oriental Yeast Co., Ltd., Tokyo, Japan) once daily. Water was provided ad libitum. The nine dogs were randomly divided into three groups: a water-treated control group and two AGE-treated groups (45 and 90 mg AGE/kg body weight once a day), with each group consisting of three dogs (two males and one female). At the time of administration, stored AGE powder was dissolved in deionized water to prepare net concentrations of 22.5 and 45 mg/ml. Forced oral doses of AGE solution in water in amounts of 2 ml/kg body weight (0, 45, and 90 mg/kg, respectively) were administered once daily approximately one hour before feeding for 12 weeks using plastic syringes.

### Clinical observation and measurement of body weight

The animals were inspected daily for their appetite, coat condition, feces characters, urine color, and behaviors before administration of AGE and an hour after it. The dogs were given 230 g of the above-mentioned dry food once daily, and the remaining amount of food was measured at 24 h after the last feeding. The dogs were weighed on the initial day of the administration and then in weeks 4, 8, and 12 after it.

### Hematology and serum biochemistry

Approximately 5.5 ml of blood was collected from the cephalic vein just before administration of AGE and then 1, 4, 8, and 12 weeks after. One ml of the blood was put into a tube with ethylenediaminetetraacetic acid dipotassium salt for hematological examinations, including CBC and blood smear examinations. Serum was obtained from approximately 4 ml of the collected blood for biochemical analysis.

The CBC, including counts of red blood cell (RBC), white blood cell (WBC), platelets (PLT), Hb concentration, hematocrit (Ht) value, mean corpuscular volume (MCV), mean corpuscular hemoglobin (MCH), and mean corpuscular hemoglobin concentration (MCHC), was determined using a hematology analyzer (XT-2000iV, Sysmex, Tokyo, Japan). Blood smears were stained with neutral red and brilliant green for the detection of reticulocytes and Heinz bodies in the erythrocytes, whereas May-Grünwald-Giemsa stain was used for the detection of eccentrocytes and any other types of poikilocytosis. Counts of reticulocytes, erythrocytes with Heinz bodies, and eccentrocytes were determined by microscopic observation. Activities of LDH, ALT, AST, ALP, and CK, as well as concentrations of TP, Alb, Glb, Glu, TG, T-Cho, BUN, Cre, T-Bil, CRP, Ca, iP, Na, K, and Cl were determined using an automated biochemical analyzer (CA-BM6050, JEOL, Tokyo, Japan).

### RNA extraction and quantitative real-time reverse transcription-polymerase chain reaction (qRT-PCR) analysis

For qRT-PCR analysis, 0.5 ml of the collected blood samples was mixed with 1.3 ml of RNAlater RNA stabilization reagent (Invitrogen, Waltham, MA, USA) and kept at − 20 °C until further use. Whole blood cell precipitate was prepared by melting and centrifugation of the stored samples. Total RNA was extracted using TRIzol reagent (Thermo Fisher Scientific, Waltham, MA, USA) and complementary DNA was synthesized using PrimeScript II (TaKaRa Bio, Shiga, Japan) according to the manufacture’s protocol. Moving on, qRT-PCR was performed using SYBR green as the detection reagent along with pairs of primers for each targeted gene (Table 3). Expressions of the *NFE2L2* and Nrf2-regulated phase II antioxidant enzyme genes: *NQO1*, *GCLM*, *HMOX1*, and *SOD2*, were measured on the basis of mRNA levels of house-keeping actin-beta (*ACTB*) gene using the comparative 2^−ΔΔCT^ method.

**Table 3:** Primers used for quantitative real-time reverse transcription polymerase chain reaction

Genes	Primers	Sequence (5′ to 3′)	NCBI Reference
Nuclear factor erythroid 2-related factor 2 (*NFE2L2*)			
	cNrf2-P1	CCCATCGGAAACCAGTGCAT	XM_014110726.1
	cNrf2-M1	CATCTACGAACGGGAATGTCTCTG	
NAD(P)H quinone dehydrogenase 1 (*NQO1*)			
	cNqo1-P1	GAAGCCGCAGACCTGGTGAT	XM_848524.4
	cNqo1-M1	GCACTCGCTCGAACCAGCCT	
Glutamate-cysteine ligase modifier subunit (*GCLM*)			
	cGCLM-P2	GCCTCCAGACTTGACTGCAT	XM_005621897.2
	cGCLM-M2	GGATGCTTTCCTGAAGGGCT	
Heme oxygenase 1 (*HMOX1*)			
	cHmox-P1	CTTTCAGAAGGGCCAGGTGAC	NM_001194969.1
	cHmox-M1	TGCTCGATCTCCTCCTCCAG	
Superoxide dismutase 2 (*SOD2*)			
	cSod2-P1	ATCGAGGAGAAGTATCTGGAGG	XM_533463.5
	cSod2-M1	CCTGAGCCTTGGACACCGAC	
Actin beta (*ACTB*)			
	cActB-P1	CGTGAGAAGATGACCCAGAT	NM_001195845.2
	cActB-M1	CGTGAGGATCTTCATGAGGTAGT	

### Statistical analyses

Statistical analyses were performed using the SPSS software (ver. 11.5.1 J, ADACS, Tokyo, Japan). Data were shown as mean ± standard deviation. Statistical significance was determined by one-way analysis of variance (ANOVA) using Dunnett’s multi-comparison procedures. Differences with *p* < 0.05 were considered statistically significant.

## Additional files

Additional file 1: The number of days when soft feces or vomiting was observed before and 1 h after the administration of AGE during the experimental period (84 days). (DOCX 19 kb)

Additional file 2: Change in body weight before and 4, 8, and 12 weeks after the administration of AGE during the experimental period. (DOCX 13 kb)

## Abbreviations

2PTS: Sodium 2-propenyl thiosulfate
AGE: Aged garlic extract
Alb: Albumin
ALP: Alkaline phosphatase
ALT: Alanine aminotransferase
ANOVA: Analysis of variance
AST: Aspartate aminotransferase
BUN: Blood urea nitrogen
Ca: Calcium
CBC: Complete blood count
CK: Creatine kinase
Cl: Chloride
Cre: Creatinine
CRP: C-reactive protein
Glb: Globulin
Glu: Glucose
Hb: Hemoglobin
Ht: Hematocrit
iP: Inorganic phosphorus
K: Potassium
Keap1: Kelch-like ECH-associated protein 1
LDH: Lactate dehydrogenase
MCH: Mean corpuscular hemoglobin
MCHC: Mean corpuscular hemoglobin concentration
MCV: Mean corpuscular volume
Na: Sodium
NPTS: Sodium *n*-propyl thiosulfate
Nrf2: Nuclear factor erythroid 2-related factor 2
PLT: Platelet
qRT-PCR: Quantitative real-time reverse transcription-polymerase chain reaction
RBC: Red blood cell (erythrocyte)
S1PC: *S*-1-propenylcysteine
SAC: *S*-allylcysteine
T-Bil: Total bilirubin
T-Cho: Total cholesterol
TG: Triglyceride
TP: Total protein
WBC: White blood cell (leukocyte)
