# Supplementary material for: Safety and efficacy of aged garlic extract in dogs: upregulation of the nuclear factor erythroid 2-related factor 2 (Nrf2) signaling pathway and Nrf2-regulated phase II antioxidant enzymes
Source: BMC Vet Res. 2018 Nov 29;14:373. doi: 10.1186/s12917-018-1699-2 (PMC6267818; doi:10.1186/s12917-018-1699-2)
Supplement: Supplementary file 2 — Change in body weight before and 4, 8, and 12 weeks after the administration of AGE during the experimental period. (DOCX 13 kb) [file 12917_2018_1699_MOESM2_ESM.docx]

Additional file 2

Change in body weight before and 4, 8, and 12 weeks after the administration of AGE during the experimental period.

| Dose (mg/kg) | n | Body weight (kg) | | | | Change*  (kg) |
| --- | --- | --- | --- | --- | --- | --- |
|  |  | Before admin. | 4 weeks  after admin. | 8 weeks  after admin. | 12 weeks  after admin. |  |
| 0 | 3 | 11.9  (0.82) | 11.4  (0.69) | 11.5  (0.69) | 11.4  (0.36) | -0.50  (0.72) |
| 45 | 3 | 11.6  (1.40) | 11.2  (1.16) | 11.4  (1.50) | 11.4  (1.57) | -0.20  (0.21) |
| 90 | 3 | 11.2  (0.72) | 10.7  (0.70) | 11.0  (0.95) | 11.0  (1.01) | -0.20  (0.35) |

Data are expressed as mean (standard deviation) (n=3).

*Change = body weight 12 weeks after the administration − body weight before the administration.
